# Impact of furosemide on mortality and the requirement for renal replacement therapy in acute kidney injury: a systematic review and meta-analysis of randomised trials

**DOI:** 10.1186/s13613-019-0557-0

**Published:** 2019-07-24

**Authors:** Łukasz J. Krzych, Piotr F. Czempik

**Affiliations:** Department of Anaesthesiology and Intensive Care, School of Medicine in Katowice, Medical University of Silesia, 14 Medyków, 40-752 Katowice, Poland

**Keywords:** Furosemide, Mortality, Renal replacement therapy, Acute kidney injury, Acute renal failure

## Abstract

**Objective:**

To examine the impact of furosemide on mortality and the need for renal replacement therapy (RRT) in adult patients with acute kidney injury (AKI) based on current evidence.

**Data sources:**

PubMed (Medline) and Embase were searched from 1998 to October 2018.

**Study selection:**

We retrieved data from randomised controlled trials comparing prevention/treatment with furosemide at any stage of AKI with alternative treatment/standard of care/placebo. The outcome was short-term mortality and the requirement for RRT, when applicable.

**Data extraction:**

Two reviewers independently extracted appropriate data. PRISMA guidelines were followed for data preparation and reporting.

**Data synthesis:**

We identified 20 relevant studies (2608 patients: 1330 in the treatment arm and 1278 in the control arm). Heterogeneity between studies was deemed acceptable, and the publication bias was low. Furosemide had neither an impact on mortality (OR = 1.015; 95% CI 0.825–1.339) nor the need for RRT (OR = 0.947; 95% CI 0.521–1.721). Furosemide had also no effect on the outcomes in strata defined by intervention strategy (prevention/treatment), AKI origin (cardio-renal syndrome, post-cardiopulmonary bypass, critical illness), control arm comparator (RRT, saline/placebo/standard of care) and its dose (< 160/≥ 160 mg) (*p* > 0.05 for all). Subjects who received furosemide with matched hydration in prevention of contrast-induced nephropathy (CIN) had a less frequent need for RRT (OR = 0.218; 95% CI 0.05–1.04; *p* = 0.055).

**Conclusions:**

Furosemide administration has neither an impact on mortality nor the requirement for RRT. Patients at risk of CIN may benefit from furosemide administration. Further well-designed RCTs are needed to verify these findings.

**Electronic supplementary material:**

The online version of this article (10.1186/s13613-019-0557-0) contains supplementary material, which is available to authorized users.

## Introduction

Acute kidney injury (AKI) constitutes a serious clinical hazard in critically ill patients. There are multiple acknowledged risk factors for AKI which can be found in the intensive care unit (ICU) setting, including sepsis, circulatory shock, trauma, and use of nephrotoxic drugs [1]. Therefore, it is not surprising that AKI develops quite frequently, affecting one in five of all hospitalised patients [2], and more than half of all patients admitted to the ICU [3]. AKI has serious sequelae: progression to chronic renal failure; increased risk and progression of multiple organ failure; increased risk of cardiovascular disease; and, finally, increased in-hospital mortality [4].

Pharmacological and non-pharmacological interventions are made for AKI treatment, including metabolic and haemodynamic stabilisation, growth factor and adenosine receptor antagonist’s administration. Unfortunately, some of these were found to have unsatisfactory results. According to KDIGO guidelines [1], diuretics should not be used for the treatment of AKI, except for fluid overload. However, this is based on low evidence (II C recommendation). RRT is the only treatment option for severe AKI.

Previously, several investigators were concerned with increased mortality in patients with AKI treated with furosemide [5, 6]. These findings were rejected by many others [7, 8]. Due to the discrepancy mentioned earlier, the impact of furosemide on mortality in AKI patients has become a subject of several systematic reviews and meta-analyses [9–13]. As all of these meta-analyses included papers published over 20 years ago, their results should be cautiously extrapolated to current clinical practice due to evident changes in the treatment of the patients at risk of AKI (e.g. those with cardio-renal syndrome, sepsis, in the perioperative period, in the ICU setting). Therefore, we attempted to verify this interesting relationship taking into account the most recent data. We also sought to investigate the possible impact of furosemide on the requirement for renal replacement therapy (RRT).

## Materials and methods

**Fig. 1:**
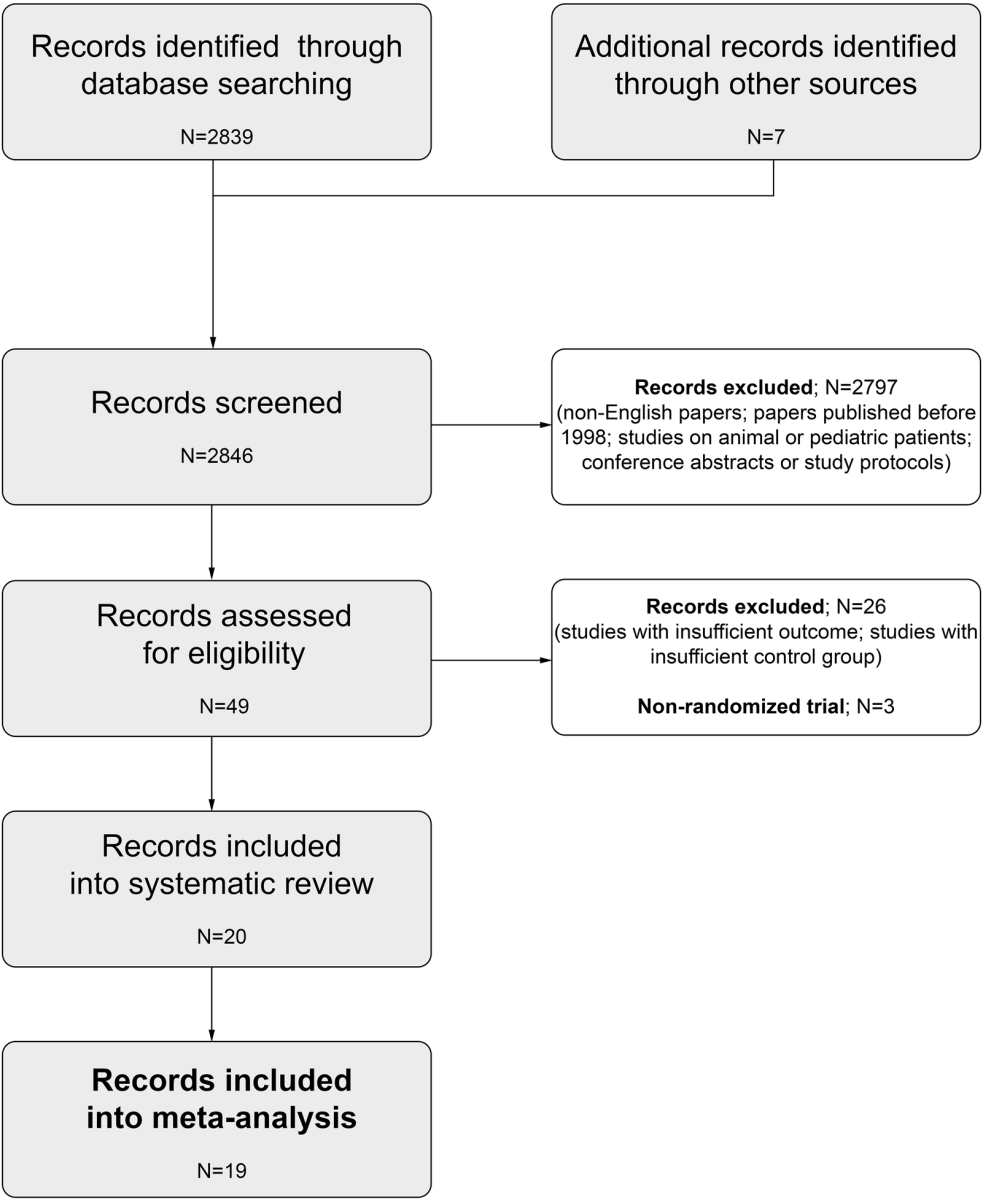
Flow diagram of the study selection process

In this systematic review, we conducted a comprehensive literature search for studies published in English from 1998 up to October 2018. We searched the electronic databases PubMed (Medline) and Embase using a pre-specified strategy (Additional file 1: Table S1). The MeSH (Medical Subject Headings) terms and key words were: furosemide, mortality, renal replacement therapy, acute kidney injury, acute renal failure and renal insufficiency. We searched for all related randomised controlled trials (RCTs) that compared prevention or treatment with furosemide, to a placebo, standard of care or RRT in adult patients with AKI, or at risk of AKI (or acute renal failure). Moreover, additional relevant studies were searched manually by checking the reference lists of identified studies or reviews. We excluded unpublished reports and conference abstracts. Two independent investigators (LJK and PFCz) screened the abstracts and/or manuscripts and extracted appropriate data. Short-term mortality was considered a primary outcome while the requirement for RRT was a secondary outcome. If the outcomes (i.e. OR; 95% CI) for individual RCTs were not revealed in the index publications, we calculated them based on raw data. Subgroup analyses defined by intervention strategy (prevention/treatment), AKI origin (cardio-renal syndrome, contrast-induced nephropathy, post-cardiopulmonary bypass, critical illness), control arm comparator (RRT, saline/placebo/standard of care) and furosemide dose (< 160/≥ 160 mg) were also performed. PRISMA guidelines were followed for appropriate data preparation and reporting [14]. A review protocol regarding this study has not been published before. Flow diagram of the study selection process is presented in Fig. 1. The quality of the trials was verified using the RoB 2.0 tool for assessing the risk of bias in randomised trials (Table 1) [15]. No disagreements occurred between the investigators on the quality of data extracted.

**Table 1:** Quality assessment of the included studies

Study	Selection bias		Performance bias	Detection bias	Attrition bias	Reporting bias	Other bias
	Random sequence generation	Allocation concealment	Blinding of participants and personnel	Blinding of outcome assessment	Incomplete outcome bias	Selective reporting	
Badawy [16]	U	U	H	L	U	L	H
Bagshaw [17]	L	L	L	L	H	L	H
Barbanti [18]	L	H	L	L	L	L	H
Bart [19]	U	U	H	L	H	H	H
Bart [20]	L	L	H	L	L	L	H
Berthelsen [21]	L	L	H	L	H	H	H
Briguori [22]	U	U	H	L	H	L	H
Cantarovich [8]	U	U	L	L	L	L	H
Costanzo [23]	U	U	H	L	U	H	H
Costanzo [24]	U	U	H	L	U	H	H
Costanzo [25]	L	L	H	L	H	H	H
Dussol [26]	L	L	H	L	U	H	H
Grams [27]	L	L	H	L	U	L	H
Hanna [28]	U	U	H	L	H	H	H
Lassnigg [7]	L	H	L	L	H	L	H
Lim [29]	L	U	L	L	H	H	H
Mahesh [30]	U	U	L	L	H	H	H
Marenzi [31]	L	U	L	L	H	H	H
Marenzi [32]	L	L	H	L	L	H	H
van der Voort [33]	U	U	L	L	L	H	H

*H* high, *L* low, *U* unknown

### Statistical analysis

Statistical analysis was performed using licensed MedCalc Statistical Software version 17.7 (MedCalc Software bvba, Ostend, Belgium; http://www.medcalc.org; 2017). We applied random and fixed effect models to verify the association between furosemide treatment and the outcomes. Odds ratios (OR) with 95% confidence intervals (95% CI) were calculated. Forest plots were drawn. We used *I*^2^ test to evaluate the magnitude of heterogeneity between studies, with values more than 50% defined as significant heterogeneity. Funnel plots were drawn to visually evaluate the potential publication bias. *P* < 0.05 was considered significant.

## Results

We identified 20 relevant studies (2608 patients: 1330 in the treatment arm and 1278 in the control arm) [7, 8, 16–33] (Table 2, Additional file 2: Table S2). There were 7 studies on AKI prevention and 13 studies describing AKI treatment with furosemide. Eight trials investigated AKI secondary to heart failure (i.e. cardio-renal syndrome), 4 described contrast-induced AKI (i.e. contrast-induced nephropathy), and 4 studies were conducted in the ICU setting (i.e. AKI of heterogeneous origin). Eighteen trials revealed data on mortality, while 8 trials presented data on the need for RRT. The quality of the papers was deemed suboptimal with the risk of bias noticeable across them (Table 1). Nineteen were included in the meta-analysis (i.e. except from the study of Lim [29]). It was possible to calculate the daily furosemide dose in 17 studies (i.e. apart from the studies of Barbanti [18], Hanna [28] and van der Voort [33]), based on mean or median values given by the authors, or based on the infusion rate and body weight of the participants. This ranged from 20 to 2500 mg, with a median value of 160 mg. A detailed description of all reviewed and included trials is presented in Supplemental Digital Content.

**Table 2:** Systematic review of studies on furosemide and the outcome

Study	Description of AKI cause	Intervention	Control arm	Mortality by crude data and OR (95% CI) and *p* value	Need for RRT by crude data and OR (95% CI) and *p* value
Badawy [16]	ADCHF	Treatment	HDF	Study group 5/20, control group 3/20; OR = 1.89 (95% CI 0.38–9.27); *p* = 0.43	NA
Bagshaw [17]	AKI (ICU patients)	Treatment	Saline	Study group 3/37, control group 5/36; OR = 0.55 (95% CI 0.12–2.48); *p* = 0.43	Study group 10/37, control group 10/36; OR = 0.96 (95% CI 0.34–2.69); *p* = 0.94
Barbanti [18]	CIN (post TAVI)	Prevention	Saline	Study group 1/56, control group 2/56; OR = 0.49 (95% CI 0.04–5.57); *p* = 0.57	NA
Bart [19]	ADCHF	Treatment	Standard of care	Study group 0/20, control group 1/20; OR = 0.32 (95% CI 0.01–8.26); *p* = 0.49	NA
Bart [20]	ADCHF	Treatment	UF	Study group 13/94, control group 16/94; OR = 0.78 (95% CI 0.35–1.73); *p* = 0.54	NA
Berthelsen [21]	AKI (ICU patients with fluid overload)	Treatment	Furosemide + CRRT	Study group 6/13, control group 2/7; OR = 2.14 (95% CI 0.3–15.36); *p* = 0.45	NA
Briguori [22]	CIN (CKD patients)	Prevention	Bicarbonate + NAC	NA	Study group 1/146, control group 6/146; OR = 0.16 (95% CI 0.19–1.35); *p* = 0.09
Cantarovich [8]	AKI (ICU + nephrology unit patients)	Treatment	Placebo	Study group 59/166, control group 50/164; OR = 1.26 (95% CI 0.79–1.99); *p* = 0.33	NA
Costanzo [23]	ADCHF	Treatment	UF	Study group 11/100, control group 9/100; OR = 1.25 (95% CI 0.5–3.14); *p* = 0.64	NA
Costanzo [24]	ADCHF	Treatment	UF	Study group 11/95, control group 9/94; OR = 1.24 (95% CI 0.49–3.14); *p* = 0.65	NA
Costanzo [25]	ADCHF	Treatment	UF	Study group 1/114, control group 2/110; OR = 0.48 (95% CI 0.04–5.35); *p* = 0.55	NA
Dussol [26]	CIN (CKD patients)	Prevention	Saline	NA	Study group 0/29, control group 0/30; OR = 1.03 (95% CI 0.2–53.83); *p* = 0.99
Grams [27]	AKI (ALI)	Treatment	Low-dose furosemide	Study group 64/169, control group 56/137; OR = 0.73 (95% CI 0.42–1.26); *p* = 0.26	Study group 45/169, control group 44/137; OR = 0.77 (95% CI 0.47–1.26); *p* = 0.29
Hanna [28]	ADCHF	Treatment	UF	Study group 4/17, control group 4/19; OR = 1.15 (95% CI 0.24–5.56); *p* = 0.86)	NA
Lassnigg [7]	AKI (post cardiac surgery)	Prevention	Saline	Study group 4/41, control group 1/40; OR = 4.22 (95% CI 0.45–39.5); *p* = 0.21	Study group 2/41, control group 0/40; OR = 5.13 (95% CI 0.24–110.2); *p* = 0.29
Lim [29]	AKI (post cardiac surgery)	Prevention	Placebo	Study group 0/40, control group 0/39; OR = 0.98 (95% CI 0.19–50.4); *p* = 0.99	NA
Mahesh [30]	AKI (post cardiac surgery)	Prevention	Saline	Study group 1/21, control group 2/21; OR = 0.47 (95% CI 0.04–5.68); *p* = 0.56	Study group 1/21, control group 0/21; OR = 3.15 (95% CI 0.12–81.74); *p* = 0.49
Marenzi [31]	CIN (CKD patients)	Prevention	Saline	Study group 1/87, control group 3/83; OR = 0.31 (95% CI 0.03–3.04); *p* = 0.31	Study group 1/87, control group 3/83; OR = 0.31 (95% CI 0.03–3.04); *p* = 0.31
Marenzi [32]	CHF	Treatment	UF	Study group 11/29, control group 7/27 OR = 1.75 (95% CI 0.56–5.45); *p* = 0.34;	NA
van der Voort [33]	AKI (ICU patients post CVVHF)	Treatment	Placebo	Study group 4/36, control group 4/35; OR = 0.97 (95% CI 0.22–4.22); *p* = 0.96	Study group 13/36, control group 7/35; OR = 2.26 (95% CI 0.77–6.6); *p* = 0.14

*ADCHF* acute decompensated chronic heart failure, *AHF* acute heart failure, *AKI* acute kidney injury, *ALI* acute lung injury, *ARF* acute renal failure, *CHF* chronic heart failure, *CIN* contrast-induced nephropathy, *CKD* chronic kidney disease, *CVVHF* continuous venovenous hemofiltration, *CRRT* continuous renal replacement therapy, *HDF* hemodiafiltration, *ICU* intensive care unit, *NA* non-applicable, *NAC* N-acetylcysteine, *OR* odds ratio, *RCT* randomised controlled trial, *TAVI* transcatheter aortic valve implantation, *UF* ultrafiltration

**Fig. 2:**
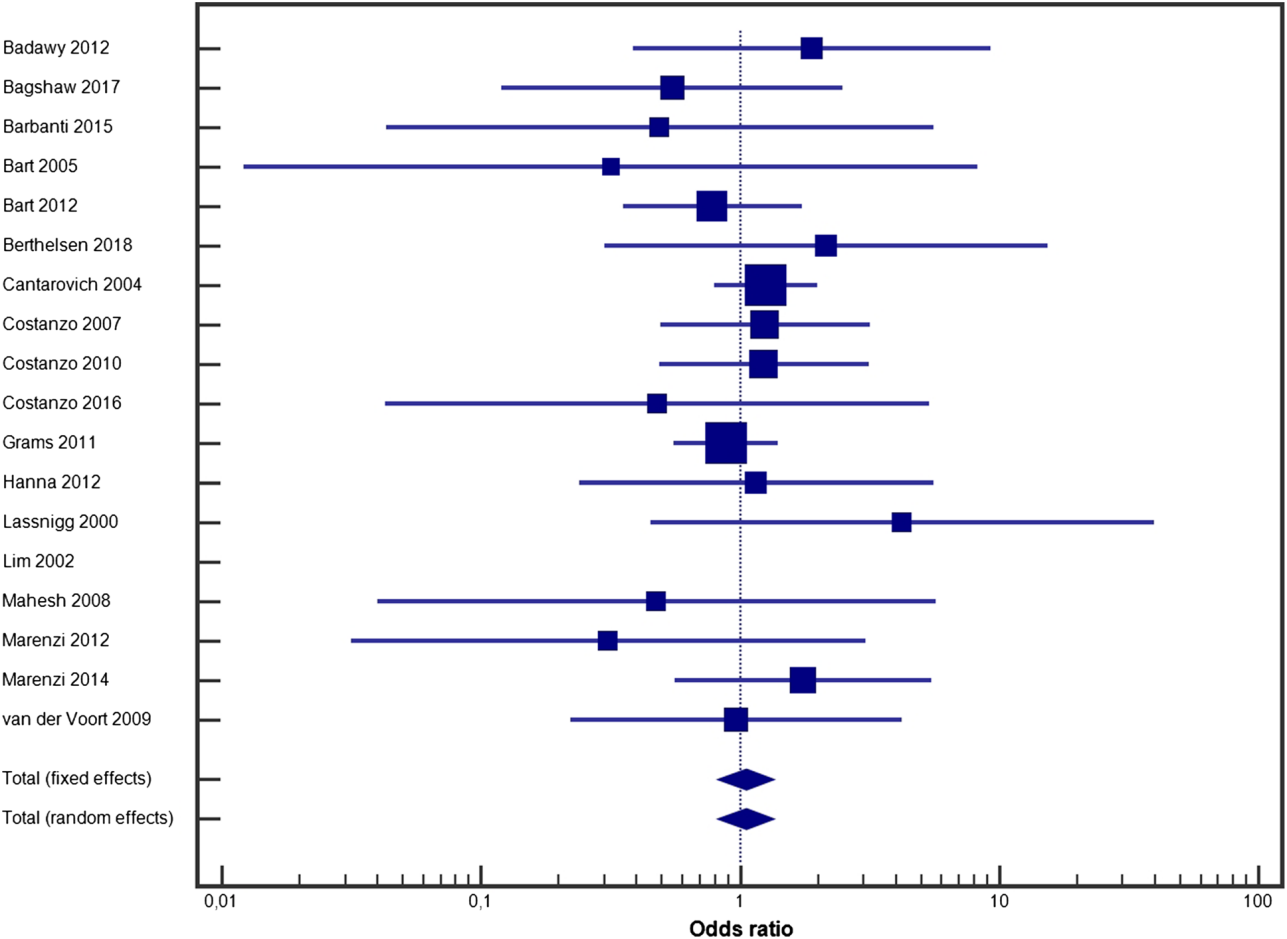
Forest plot for furosemide use and mortality

**Fig. 3:**
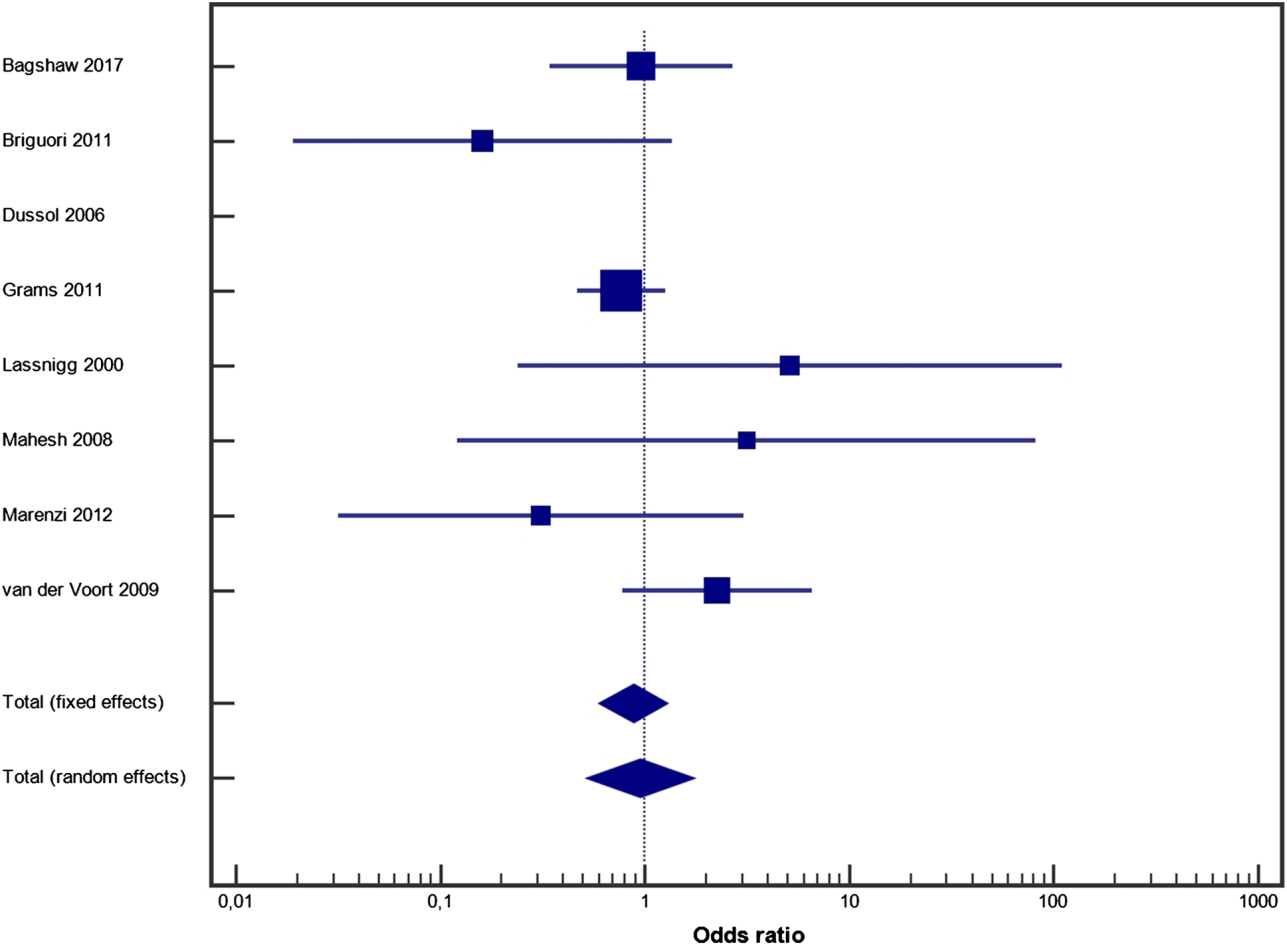
Forest plot for furosemide use and the requirement for renal replacement therapy

Furosemide had no impact on mortality (random effect model OR = 1.051, 95% CI 0.825–1.339, *p* = 0.4; fixed effect model OR = 1.051, 95% CI 0.827–1.334, *p* = 0.4) (Fig. 2). Heterogeneity between studies was low (*I*^2^ = 0%, 95% CI 0–10.4%, *p* = 0.9), and publication bias was low (Additional file 3: Figure S1A). Furosemide had no impact on the need for RRT (random effect model OR = 0.947, 95% CI 0.521–1.721, *p* = 0.2; fixed effect model OR = 0.878, 95% CI 0.602–1.281, *p* = 0.7) (Fig. 3). Heterogeneity was acceptable (*I*^2^ = 28.62%, 95% CI 0–69.29%, *p* = 0.2), and publication bias was low (Additional file 4: Figure S1B).

The results of additional analyses are shown in Table 3. Irrespective of intervention strategy (prevention/treatment), AKI origin (cardio-renal syndrome, post-cardiopulmonary bypass, critical illness), control arm comparator (RRT, saline/placebo/standard of care) and furosemide dose (< 160/≥ 160 mg), there was no effect of furosemide either on mortality or the need for RRT. However, subjects who received furosemide with matched hydration in the prevention of CIN had a less frequent need for RRT (by 79%, with borderline significance).

**Table 3:** Subgroup analyses

Subgroup analysis	Description	Mortality		Requirement for RRT	
		Fixed effect model	Random effect model	Fixed effect model	Random effect model
By intervention	Prevention	OR = 0.852 (95% CI 0.303–2.395), *p* = 0.7	OR = 0.780 (95% CI 0.235–2.590), *p* = 0.7	OR = 0.584 (95% CI 0.210–1.625), *p* = 0.3	OR = 0.678 (95% CI 0.133–3.450), *p* = 0.6
	Treatment	OR = 1.063 (95% CI 0.832–1.359), *p* = 0.6	OR = 1.065 (95% CI 0.831–1.364), *p* = 0.6	OR = 0.904 (95% CI 0.625–1.413), *p* = 0.8	OR = 1.037 (95% CI 0.568–1.894), *p* = 0.9
By AKI aetiology	Cardio-renal syndrome	OR = 1.108 (95% CI 0.734–1.674), *p* = 0.6	OR = 1.114 (95% CI 0.733–1.692), *p* = 0.6	–	–
	CIN	OR = 0.381 (95% CI 0.07–1.996), *p* = 0.2	OR = 0.385 (95% CI 0.07–2.031), *p* = 0.3	OR = 0.211 (95% CI 0.04–0.99),* p* = 0.049	OR = 0.218 (95% CI 0.05–1.04),* p* = 0.055
	Post-CPB	OR = 1.688 (95% CI 0.388–7.345), *p* = 0.5	OR = 1.5115 (95% CI 0.178–12.869), *p* = 0.7	OR = 4.147 (95% CI 0.449–38.27), *p* = 0.2	OR = 4.075 (95% CI 0.437–38.02), *p* = 0.2
	Critical illness (ICU patients)	OR = 1.039 (95% CI 0.765–1.411), *p* = 0.8	OR = 1.039 (95% CI 0.764–1.413), *p* = 0.8	OR = 0.940 (95% CI 0.625–1.413), *p* = 0.8	OR = 1.037 (95% CI 0.568–1.894), *p* = 0.9
By control arm treatment	RRT	OR = 1.169 (95% CI 0.778–1.757), *p* = 0.4	OR = 1.169 (95% CI 0.775–1.766), *p* = 0.4	–	–
	Saline/placebo/standard of care	OR = 0.618 (95% CI 0.304–1.256), *p* = 0.2	OR = 0.626 (95% CI 0.197–1.994), *p* = 0.4	OR = 1.397 (95% CI 0.736–2.652), *p* = 0.3	OR = 1.393 (95% CI 0.711–2.730), *p* = 0.3
By daily furosemide dose	< 160 mg	OR = 0.931 (95% CI 0.651–1.329), *p* = 0.7	OR = 0.923 (95% CI 0.642–1.326), *p* = 0.7	OR = 0.727 (95% CI 0.466–1.134), *p* = 0.2	OR = 0.703 (95% CI 0.315–1.568), *p* = 0.4
	≥ 160 mg	OR = 1.194 (95% 0.848–1.680), *p* = 0.3	OR = 1.201 (95% CI 0.850–1.695), *p* = 0.3	–	–

*AKI* acute kidney injury, *CI* confidence interval, *CIN* contrast-induced nephropathy, *CPB* cardiopulmonary bypass, *ICU* intensive care unit, *OR* odds ratio, *RRT* renal replacement therapy

## Discussion

In this systematic review of 20 RCTs covering over 2600 patients with AKI, or at risk of AKI, we found that furosemide had an impact neither on mortality nor on the requirement for RRT. Furosemide had no effect on the outcomes regardless of intervention strategy, AKI origin, control arm comparator and furosemide dose. Only patients at risk of CIN may benefit from furosemide administration.

These observations are comparable with the available data. In the most recent meta-analysis, Bove et al. [13] revealed no association between furosemide and mortality, the need for RRT, length of hospital stay and worsening of AKI. The authors found significant improvement in survival of patients receiving furosemide as a preventive measure (OR = 0.62; 95% CI 0.41–0.94). It needs to be underlined that they included 8 studies published over 20 years ago and compared intermittent furosemide administration (only) with any comparator (including continuous furosemide infusion) in subjects with or at risk of AKI. In their meta-analysis published in 2006, Ho and Sheridan [9] also found no impact of furosemide, either on mortality or on the need for RRT, regardless of the fact whether it was used to prevent acute deterioration of renal function or treat acute renal failure. However, they included only 9 RCTs describing ARF patients, while only 3 of them investigated the requirement dialysis. Moreover, Bagshaw et al. [10] in 2007 described no effect of furosemide on mortality and renal recovery. In their study, loop diuretics were associated with a shorter duration of RRT (by 1.4 days), a shorter time to spontaneous decline in serum creatinine level (by 2.1 days) and a greater increase in urine output from the baseline (OR = 2.56; 95% CI 1.35–4.85). However, they analysed 5 RCTs only, 4 of which were published over 20 years ago, and all of which were of very low quality. Last but not least, Cheng et al. [34] in their meta-analysis of 7 RCTs, revealed comparable mortality between patients with decompensated heart failure receiving heamofiltration or diuretics (OR = 0.95; 95% CI 0.58–1.55).

Our findings are also in line with the results of Putzu et al. [35] who evaluated whether the administration of furosemide with matched hydration using the RenalGuard System might have reduced the incidence of CIN. Based on 4 trials, they found the beneficial impact of furosemide use on CIN occurrence (OR = 0.31; 95% CI 0.19–0.50), as well as the need for RRT (OR = 0.19; 95% CI 0.05–0.79).

From a clinical point of view, our observations are of particular importance to practitioners as, based on experimental data, furosemide may have some detrimental effects in patients with or at risk of AKI **[**5, 6, 36]. This observation plays also a key role in determining the necessity of early initiation of RRT in critically ill subjects with deterioration of renal function and/or fluid overload. This is in line with the above-mentioned observations of Cheng et al. [34].

Fluid overload is frequently found in AKI patients in the ICU setting. Fluid excess of at least 10% is associated with increased mortality and morbidity, including pulmonary oedema, cardiac failure, delayed wound healing, tissue breakdown and impaired bowel function [37, 38]. Although there are several methods of evaluating fluid status, most of them are fairly inaccurate [39, 40]. Moreover, the optimisation and management of fluid therapy remains difficult [41–43]. Diuretics are considered a reasonable first-line treatment but only in responsive subjects [1]. When selecting a diuretic, clinicians should consider evidence-based indications, possible adverse effects, compatibility, pharmacokinetics and other issues of particular diuretics [36]. Diuretics are not recommended to treat AKI per se but are suggested to treat volume overload [37]. According to current data, it is still difficult to predict which patients will respond to diuretics and which patients will benefit from early initiation of RRT.

The severity of AKI alters both the pharmacokinetics and pharmacodynamics of furosemide [40], making appropriate dosing of this agent difficult. The requirement for increased dosing of loop diuretics is associated with worse AKI prognosis in patients with congestive heart failure [44] and in other groups of critically ill subjects [45]. The use of high-dose furosemide to convert oliguric to non-oliguric AKI may exacerbate injury to the kidneys (increased markers of oxidative stress), especially in hypovolaemic patients with reduced renal perfusion [46].

Based on our results, implementation of RRT should be considered at an early stage of critical illness in patients with fluid overload, as well as those with AKI and concomitant indications for immediate extracorporeal treatment [47]. The effect of RRT modality seems to be of lower importance [48, 49].

### Study limitations

Firstly, we focused on trials published only in the last 20 years. All previous meta-analyses took into account data published before 1998. Although this subjective decision may cause publication bias, one ought to remember that clinical practice has improved markedly: currently we do not use such high doses of furosemide due to its side effects, while the pharmacological and non-pharmacological treatment of many co-existing acute conditions in critically ill patients has changed (acute heart failure treatment, antimicrobial treatment, perioperative care, fluid therapy, etc.). In addition, an understanding of the role of RRT in critical illness has led to its earlier implementation. Secondly, almost all data from the ICU setting compared furosemide with alternative pharmacological treatment (usually a placebo). So far, no single randomised trial has reported a comparison between furosemide and any modality of RRT in this heterogeneous group of critically ill patients. We can only extrapolate data from patients with isolated AKI due to acute decompensated heart failure to current ICU practice. Thirdly, as there are newer intravenous diuretics available on the market, including torasemide and aldactone, further research is needed to investigate their effect (on its own or in combination with furosemide) on mortality in AKI. Finally, our observations need further investigations. In the era of numerous non-renal indications for RRT, which are usually considered in the mixed population of ICU subjects, our results may be inadequate. Moreover, the quality of previously published RCTs remains suboptimal, while the heterogeneity of the populations recruited in earlier trials constitutes an important limitation in generalising results.

## Conclusions

Based on our meta-analysis of RCTs, furosemide administration has no impact either on mortality or on the requirement for RRT, regardless of intervention strategy, AKI origin, control arm comparator and furosemide dose. However, patients at risk of CIN may benefit from furosemide treatment, if proper hydration is guaranteed beforehand. Further well-designed RCTs are needed in order to verify these findings.

## Additional files

**Additional file 1: Table S1.** Search strategy.

**Additional file 2: Table S2.** Detailed characteristics of included studies.

**Additional file 3: Figure S1A.** Funnel plot for studies on mortality.

**Additional file 4: Figure S1B.** Funnel plot for studies on the requirement for renal replacement therapy.

## Data Availability

All data generated or analysed during this study are included in this published article [and its supplementary information files].
