# Supplementary material for: Impact of furosemide on mortality and the requirement for renal replacement therapy in acute kidney injury: a systematic review and meta-analysis of randomised trials
Source: Ann Intensive Care. 2019 Jul 24;9:85. doi: 10.1186/s13613-019-0557-0 (PMC6656832; doi:10.1186/s13613-019-0557-0)
Supplement: Supplementary file 1 — Additional file 1: Table S1. Search strategy. [file 13613_2019_557_MOESM1_ESM.docx]

Table S1. Search strategy

| #1. PubMed search on 10/20/2018 using ‘MeSH terms’ & ‘best match’ strategy: | N= 306 |
| --- | --- |
| (furosemide AND acute kidney injury AND mortality) OR (furosemide AND renal insufficiency AND mortality) OR (furosemide AND acute kidney injury AND renal replacement therapy) OR (furosemide AND renal insufficiency AND renal replacement therapy) |  |
| #2. PubMed search on 10/20/2018 using ‘key words’ & ‘best match’ strategy: | N= 1111 |
| (furosemide AND acute kidney injury AND mortality) OR (furosemide AND acute renal failure AND mortality) OR (furosemide AND renal insufficiency AND mortality) OR (furosemide AND acute kidney injury AND renal replacement therapy) OR (furosemide AND acute renal failure AND renal replacement therapy) OR (furosemide AND renal insufficiency AND renal replacement therapy) |  |
| #3. Embase search on 10/20/2018 using ‘all fields’ algorithm: | N= 1422 |
| (furosemide AND acute kidney failure AND mortality) OR (furosemide AND kidney injury AND mortality) OR (furosemide AND acute kidney failure AND renal replacement therapy) OR (furosemide AND kidney injury AND renal replacement therapy) |  |

MeSH – Medical Subject Headings
