# Supplementary material for: Impact of furosemide on mortality and the requirement for renal replacement therapy in acute kidney injury: a systematic review and meta-analysis of randomised trials
Source: Ann Intensive Care. 2019 Jul 24;9:85. doi: 10.1186/s13613-019-0557-0 (PMC6656832; doi:10.1186/s13613-019-0557-0)
Supplement: Supplementary file 2 — Additional file 2: Table S2. Detailed characteristics of included studies. [file 13613_2019_557_MOESM2_ESM.docx]

Table S2. Detailed characteristics of included studies

| Study | Patient population | AKI description (cause of AKI) | Intervention | Study group and furosemide dose | Control group and control arm | Outcome: mortality | Outcome: need for RRT | Comments (risk of bias) |
| --- | --- | --- | --- | --- | --- | --- | --- | --- |
| Badawy 2012 | patients with acute decompensated CHF with fluid overload admitted to the ICU (NYHA III and IV), all had lower extremity edema and at least one additional feature of CHF | CHF | Treatment | n=20,  loading dose 1mg/kg then continuous infusion 20mg/h increased if required to maintain UO of > 1mL/kg/h | n=20,  HDF, effluent flow 35mL/kg/h, ratio of dialysate to replacement fluid 1:1, blood flow >150 mL/min, ultrafiltration not exceeding 200 mL/h, UFH anticoagulation with ACT monitoring | 30-day mortality: study group 5/20,  control group 3/20 | NA | small sample size, treatment not blinded |
| Bagshaw 2017 | adult ICU patients with mean APACHE II score of 26.6±7.8, 79.5% were medical admissions, 90.4% received mechanical ventilation, 61.6% received vasoactive drugs | early AKI (minimum RIFLE R category ), AKI in patients with ≥2 SIRS criteria, often patients with sepsis | Treatment | n=37,  loading dose 0.4mg/kg followed by a continuous infusion starting at 0.05mg/kg/h to achieve a target UO of 1-2mL/kg/h, maximum infusion rate 0.40 mg/kg/h, infusion continued for a minimum 24 hrs | n= 36,  saline bolus plus continuous infusion | hospital death: study group 3/37,  control group 5/36  p=0.5 | study group 10/37, control group 10/36 p=0.9 | small trial terminated prematurely, risk of type I error, suboptimal statistical power, high rate of protocol deviations |
| Barbanti 2015 | 112 elderly patients (mean age of 80.6 ± 5.1 years) undergoing elective transcatheter aortic valve replacement (at risk of AKI), data retrieved from the Replace Registry, patients on chronic dialysis and with RF< 30% excluded | CI-AKI caused by radiologic contrast used intraoperative- TAVI procedure | Prevention | n=56, furosemide-induced diuresis with matched isotonic saline intravenous hydration using the RenalGuard System, prehydration with isotonic saline for 10 hrs before TAVI, then Renal Guard System started- priming of 250 mL (150mL in patients with EF < 30%), then furosemide given, loading dose of 0.25mg/kg to achieve urine output of > 300mL/h, additional doses of furosemide permitted if UO < 300mL/h | n=56, continuous infusion of isotonic saline (commencement 12 h prior TAVI and continued for 6h after the procedure) at a rate of 1 mL/kg/h (in patients with EF < 30% infusion rate reduced to 0.5 mL/kg/h) matched with urine output | in-hospital death: study group 1/56; control group 2/56; p=0.537 | no case of in-hospital renal failure requiring dialysis was reported, although 1/56 in the control group developed AKI stage 3 | single-centre study; open-label trial |
| Bart 2005 | acute decompensated CHF, at least 2+ edema of lower extremities and at least one additional features, population from UNLOAD trial | ADHF | Treatment | n=20, median dose 160 mg/d | n=20, UF (single 8h session with minimally-invasive device System 100, ultrafiltration dose up to 500mL/h) + furosemide (median dose 80mg/d)- withheld for the duration of UF | 30-day mortality: study group 0/20, control group 1/20 | NA | small sample size |
| Bart 2012 | acute decompensated heart failure, CARRESS-HF trial, patients with at least 2+ peripheral edema and one additional feature | ADHF | Treatment | n=94, stepped diuretic therapy, dose adjusted to maintain UO of 3-5L/d, median dose 120 (80-160) mg/d | n=94, UF (Aquadex System 100) 200mL/h + furosemide (withheld for the duration of UF) median dose 120 (80-240) mg/d | study group 13/94, control group 16/94, p=0.55 | NA | not blinded |
| Berthelsen 2018 | intensive care patients with AKI, FFAKI feasibility trial | AKI-ICU, fluid accumulation of more than 10% ideal bodyweight, moderate and high risk Aki according to Renal Recovery Score (RRS) | Treatment | n=13, cumulative median (IQR) furosemide dose (mg/kg) 2.0 (0.0-13.0) | n=7, UF( in case of anuria or inadequate diuretic response) + FURO (loading dose 40mg/kg followed by infusion titrated to a maximum dose of 40mg/h), treatment adjusted every 8 hrs to achieve a net negative fluid balance of at least 1 mL/kg IBW/h | 90-day mortality: study group 6/13, control group 2/7 | NA | stopped prematurely due to a low inclusion rate, important differences between groups at baseline, unblinded |
| Briguori 2011 | patients with an eGFR ≤ 30 mL/min/1.73 m2 and or a risk score ≥ 11 scheduled for coronary and/or peripheral angiography and/or angioplasty | CI-AKI | Prevention | n=146, hydration with saline (priming 250mL/150mL) + NAC (1500mg in 1L saline during preprocedural, intraprocedural, postprocedural phase) + furosemide (all controlled by RenalGuard System) for the time of procedure and for 4 hrs after procedure, loading dose of 0.25mg/kg to achieve urine output of > 300mL/h | n=146, hydration with 154 mEq/L sodium bicarbonate in D5W solution according to protocol (initial bolus 3mL/kg/h for at least 1 h, then 1 mL/kg/h during procedure and for 6 hrd after) + NAC (1200mg orally BID day before and on the day+ NAC 1200mg diluted in 100 mL normal saline iv during the procedure) | NA | 1/146 in study group; 6/146 in control group; p=0.056 | open-label study; powered on CI-AKI but not on dialysis and death (hard clinical end points); different doses and routes of administration of NAC in study and control group |
| Cantarovich 2004 | 338 with acute renal failure requiring dialysis (urea > 30 mmol/L, oligo-anuria for 48 hrs, uremic syndrome- French practice guidelines) from 13 ICUs and 10, nephrology wards, all patients received furosemide at the dose of 15mg/kg over 4 hrs in a screening period | ARF requiring dialysis; sepsis frequent (72/166 in study group; 54/164 in control group; p=0.055) | Treatment | n=16, 25mg/kg/d iv (maximum 2g/d) when tolerated iv infusions replaced by oral administration (35mg/kg/d; maximum 2.5g/d) | n=166 | 59/166 in study group; 50/164 in control group | NA | furosemide test dose may have contributed to relaunch diuresis in some patients in control group; baseline differences present despite randomization |
| Costanzo 2007 | hypervolemic heart failure patients, with peripheral edema 2+ and one additional feature, UNLOAD trial | ADHF | Treatment | n=100, average dose 181 ± 121 mg/d of furosemide | n=100, UF, Aquadex System 100, up to 500mL/h of utrafiltration, UFH anticoagulation according to ACT or aPTT | study group 11/100, control group 9/100 | NA | unblinded, treatment targets for diuretics and ultrafiltration not prespecified |
| Costanzo 2010 | patients hospitalized for heart failure with ≥ signs of hypervolemia | ADHF | Treatment | n=68 (bolus diuretic), n=32 (continuous diuretic), average furosemide equivalent dose 316 ± 213 mg/d (bolus group) and 335 ± 325 mg/d (continuous infusion group) | UF, Aquadex System 100, average rate of UF 141 mL/h for 12.3 ± 12 hrs | study group 11/95, control group 9/94 | NA | unblinded |
| Costanzo 2016 | primary diagnosis: acute decompensated congestive heart failure, AVOID-HF trial, all patients on fluid (1500mL/d) and salt (1.5g) restriction | ADHF | Treatment | n=114, adjustable intravenous loop diuretic, average daily dose of furosemide-equivalent dose 271.26 ± 263.06 for an average 100 ± 78 hrs | n=110, UF, Aquades FlexFlow System, average UF rate 138 ± 47 mL/h for an average 80 ± 53 h | hospital mortality: study group 1/114, control group 2/110; 90-day mortality: study group 14/114, control group 17/110 | NA | untimely termination by the sponsor |
| Dussol 2006 | patients at mean age of 64 ± 14 years, with chronic kidney disease (GFR 37 ± 12 mL/min/1.73 m2) (native kidney 80% and transplant kidney 20%) undergoing various radiological procedures | CRF- CI-AKI | Prevention | n=29; hydration with 0.9% saline (15mL/kg for 6 hrs prior procedure) + furosemide , 3mg/kg iv after procedure | n=30; hydration with 0.9% saline (15mL/kg for 6 hrs prior procedure) | NA | 0/29 in study group; 0/30 in control group | the study was initially powered to demonstrate superiority of theophylline or furosemide over intravenous saline hydration |
| Grams 2011 | patients with AKI (AKIN criteria) and AKI (mainly due to pneumonia and sepsis), all patients received furosemide | stage 1 AKI approximately 60%, stage 3 - 20-30% | Treatment | n=169; restrictive fluid strategy patients (FACTT study), 80mg/d | n=137; Low dose of furosemide (23 mg/d) | 60-day mortality: 64/169 in study group; 56/137 in control group; p=0.59 | 45/169 in study group; 44/137 in control group; p=0.29 | lack of premorbid creatinine concentration |
| Hanna 2012 | patients with acute decompensated heart failure admitted to ICU for hemodynamically guided therapy, NYHA III/IV | ADHF | Treatment | n=17, intravenous diuretic at doses and frequencies designated by the treating physician | n=19, UF, UF rate 400 mL/h for 6 hrs then 200 mL/h, blood flow rate 200-300 mL/min via central venous catheter, UFH anticoagulation | study group 4/17, control group 4/19, p=0.8584 | NA | small number of patients, unable to complete enrollment in the time period for the trial, no protocol for conventional diuretic therapy (study group) |
| Lassnigg 2000 | cardiac surgery patients (mainly CABG) with normal renal function (creatinine < 2mg/dL) undergoing elective procedures | AKI post CPB | Prevention | n=41,  0.5 mcg/kg/min starting at beginning of surgery and continuing for 48 hrs or until discharge from ICU, infusion discontinued when UO > 2000 mL in 4 hrs | n=40, isotonic sodium chloride | hospital mortality: 4/41 in study group; 1/40 in control group | 2/41 in study group; 0/40 in control group |  |
| Lim 2002 | patients undergoing primary elective CABG |  | Prevention | n=40,  40mg of furosemide+ 5mg of amiloride | n=39, placebo | study group 0/40; control group 0/39 | NA | unable to assess whether results were due to furosemide or amiloride |
| Mahesh 2008 | high-risk cardiac surgical population (pre-existing renal dysfunction, diabetes, impaired left-ventricular function, complicated procedures with prolonged CPB) | AKI post cardiac surgery | Prevention | n=21,  4mg/hstarted at induction of anestehsia and continues for 12 h following surgery | n=21, saline 2mL/h | study group 1/21 (MI); control group 2/21 (stroke, septicemia), p=0.99 | study group 1/21; control group 0/21, p=0.99 | underpowered due to small number of patients in each group |
| Marenzi 2012 | patients with chronic kidney disease (eGFR < 60 mL/min/1.73 m2 using modified formula by Levey) undergoing elective and urgent coronary procedures, MYTHOS study | CKD- CI-AKI | Prevention | n=87, furosemide-induced diuresis and matched hydration treatment with isotonic saline provided by RenalGuard System,  0.5 mg/kg iv initial bolus, additional doses given during the treatment (up to cumulative dose 2mg/kg) | n=83, isotonic saline infusion at a rate of 1mL/kg/h (o.5mL/kg/h in patients with LVEF < 40%) for 12 h before and after procedure | in-hospital death: study group 1/87; control group 3/83 | study group 1/87; control group 3/83 | single-center study, study was unblinded, treatment protocol in the study group arbitrarily predetermined |
| Marenzi 2014 | acute decompensated congestive heart failure with fluid overload, CUORE trial | CHF | Treatment | n=29,  dose at the discretion of experienced physician, baseline mean dose 153 ± 115 mg/d | n=27, UF (single or double session) up to cumulative fluid removal of > 2 L + diuretic used before randomization (baseline mean dose 194 ± 175 mg/d) | study group 11/29, control group 7/27 | NA | small sample size, treatment not blinded |
| van der Voort 2009 | mechanically ventilated patients who have been treated with CVVHF due to ARF | recovery phase of AKI after RRT | Treatment | n=36,  continuous infusion 0.5mg/kg/h | n=35 | study group 4/36, control group 4/35 | study group 13/36, control group 7/35 | small sample size, study group had a higher age and SOFA score |

ADCHF – acute decompensated chronic heart failure, AHF – acute heart failure, AKI – acute kidney injury, ALI – acute lung injury, ARF – acute renal failure, CHF – chronic heart failure, CIN – contrast-induced nephropathy, CKD – chronic kidney disease, CVVHF – continuous venovenous hemofiltration, CRRT – continuous renal replacement therapy, HDF – hemodiafiltration, ICU – intensive care unit, NA – non applicable, NAC – N-acetylcysteine, OR – odds ratio, RCT – randomised controlled trial, TAVI – transcatheter aortic valve implantation, UF – ultrafiltration
