# Supplementary figures and images for: Impact of furosemide on mortality and the requirement for renal replacement therapy in acute kidney injury: a systematic review and meta-analysis of randomised trials
Source: Ann Intensive Care. 2019 Jul 24;9:85. doi: 10.1186/s13613-019-0557-0 (PMC6656832; doi:10.1186/s13613-019-0557-0)

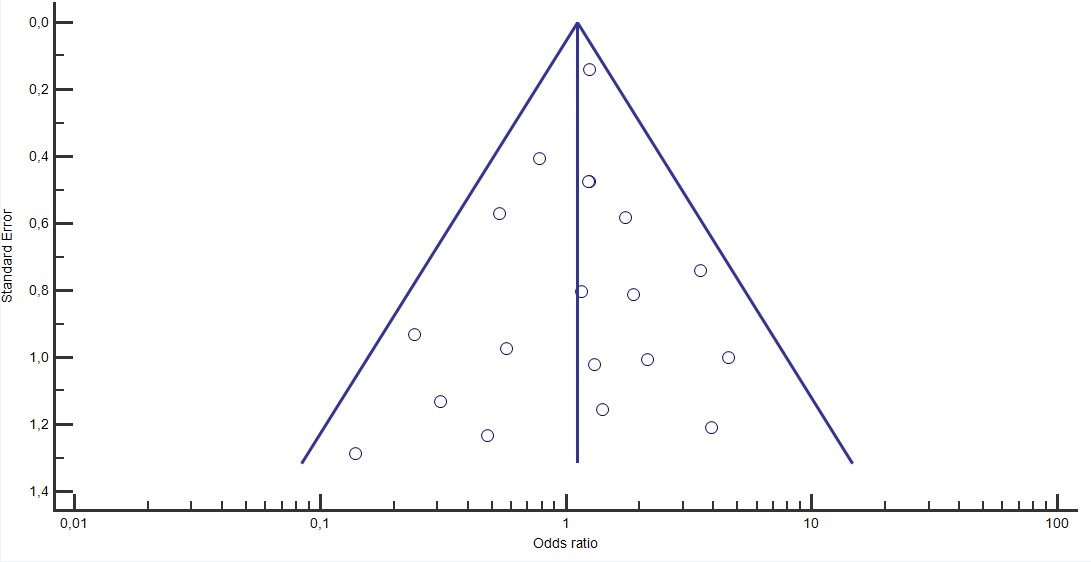

Supplement: Supplementary file 3 — Additional file 3: Figure S1A. Funnel plot for studies on mortality. [file 13613_2019_557_MOESM3_ESM.jpg]

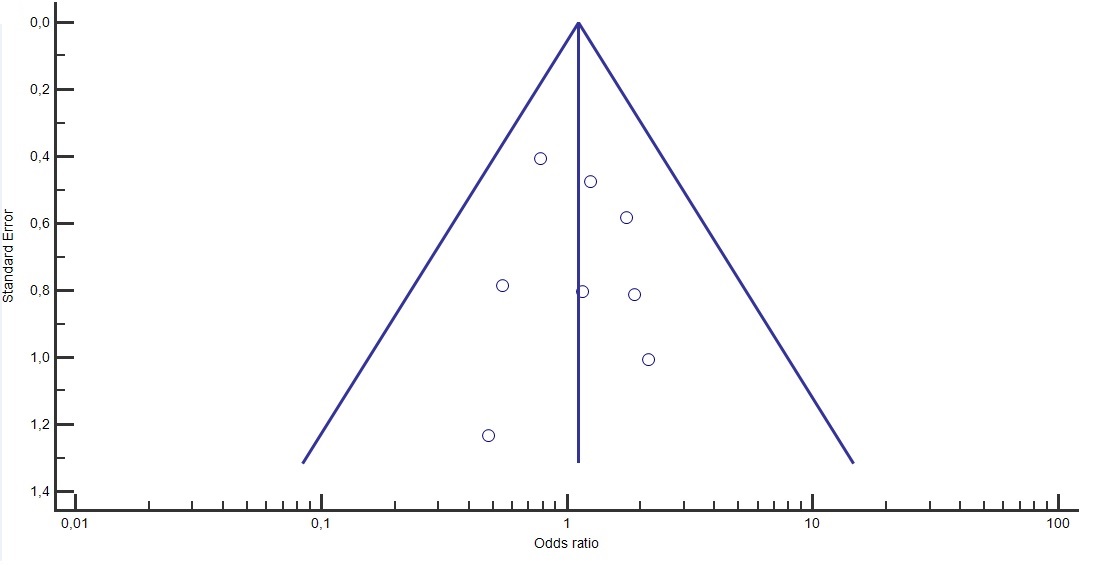

Supplement: Supplementary file 4 — Additional file 4: Figure S1B. Funnel plot for studies on the requirement for renal replacement therapy. [file 13613_2019_557_MOESM4_ESM.jpg]
